# Supplementary material for: Dynamic transcriptomic profiles of zebrafish gills in response to zinc supplementation
Source: BMC Genomics. 2010 Oct 11;11:553. doi: 10.1186/1471-2164-11-553 (PMC3091702; doi:10.1186/1471-2164-11-553)
Supplement: Additional file 2 — Interactive Direct Interaction Network representing the molecular interactions between zinc, copper, iron, calcium and proteins encoded by transcripts changed by zinc supplementation. Mini web-site containing index.html and hyperlinked pages in subdirectory describing a Direct Interaction Network automatically generated based on curated interactions contained within the proprietary PathwayArchitect database. Ovals represent proteins and the circles symbolize metal ions. Objects are coloured by their abundance in zebrafish at the time-point they were significantly different from the control is a scale from -4 fold (dark green) to +4 fold (dark red). Where significant differences were found at more than one time-point, the colour overlay shows expression at the first instance. Dark blue squares denote 'binding', and light blue squares 'expression'; green squares stand for 'regulation', green diamonds for 'metabolism', and green circles for 'promoter binding'. Arrow heads indicate directionality of the interaction where annotated. All nodes and edges can be further interrogated by selecting the relative area of the image. [file 1471-2164-11-553-S2.zip › PathwayArchitect Zn xs DIN/426880.html]

# BINDING:

|  |  |
| --- | --- |
| Type | BINDING |
| Effect | None |


---

|  |  |
| --- | --- |
| Score | 0 |


---

|  |  |
| --- | --- |
| Reference Count | 7 |


---

|  |  |
| --- | --- |
| Mechanism | Unknown |


---

|  |  |
| --- | --- |
| Reference:0 || Sentence | "We previously indicated that nuclear matrix proteins (NP/NMP4) interact with the rat type I collagen alpha1(I) polypeptide chain (COL1A1) promoter at two poly(dT) sequences (sites A and B) and bend the DNA." |
| PMID | 11149472 |
| Year | 2001 |
| Species | Rat |
| Journal | J Bone Miner Res |
| RefScore | 0 |
| Source | PArchNLP |
  |
|  |  |


---

|  |  |
| --- | --- |
 Reference:1 || Sentence | "Here, our objective was to determine whether NP/NMP4-COL1A1 binding influences promoter activity and to clone NP/NMP4." |
| PMID | 11149472 |
| Year | 2001 |
| Species | Rat |
| Journal | J Bone Miner Res |
| RefScore | 1 |
| Source | PArchNLP |
  ||  |  |


---

|  |  |
| --- | --- |
 Reference:2 || Sentence | "These clones expressed proteins with molecular weights and COLIA1 binding activity similar to NP/NMP4." |
| PMID | 11149472 |
| Year | 2001 |
| Species | Rat |
| Journal | J Bone Miner Res |
| RefScore | 0 |
| Source | PArchNLP |
  ||  |  |


---

|  |  |
| --- | --- |
 Reference:3 || Sentence | "Antibodies to these proteins disrupted native NP/NMP4-COL1A1 binding activity." |
| PMID | 11149472 |
| Year | 2001 |
| Species | Rat |
| Journal | J Bone Miner Res |
| RefScore | 0 |
| Source | PArchNLP |
  ||  |  |


---

|  |  |
| --- | --- |
 Reference:4 || Sentence | "Parathyroid hormone increased NP/NMP4 binding to both poly(dT) elements and decreased COL1A1 mRNA in the osteosarcoma cells." |
| PMID | 9581872 |
| Year | 1998 |
| Species | Rat |
| Journal | J Cell Biochem |
| RefScore | 1 |
| Source | PArchNLP |
  ||  |  |


---

|  |  |
| --- | --- |
 Reference:5 || Sentence | "NP/NMP4-COL1A1 promoter interactions may represent a molecular pathway by which osteoblast structure is coupled to COL1A1 expression." |
| PMID | 9581872 |
| Year | 1998 |
| Species | Rat |
| Journal | J Cell Biochem |
| RefScore | 1 |
| Source | PArchNLP |
  ||  |  |


---

|  |  |
| --- | --- |
 Reference:6 || Sentence | "Some NP/NMP4 isoforms bind to the rat type I collagen alpha1(I) polypeptide chain promoter in the manner of architectural transcription factors and alter basal transcription in osteoblast-like cells (Thunyakitpisal et al. in review)." |
| PMID | 10972987 |
| Year | 2000 |
| Species | Human |
|  | Rat |
| Journal | J Cell Biochem |
| RefScore | 1 |
| Source | PArchNLP |
  |


---

|  |  |
| --- | --- |
